# Spinal meningeal melanocytoma in a 5-year-old child: a case report and review of literature

**DOI:** 10.1186/s41983-018-0017-z

**Published:** 2018-05-17

**Authors:** Ahmed M. Salah El-Din, Hashem M. Aboul-Ela, Mohamed F. Alsawy, Ahmed Koheil, Ahmed H. Ashry

**Affiliations:** 1 Neurosurgery Department, Faculty of Medicine, Cairo University, Cairo, Egypt; 2 Neurosurgery Department, Faculty of Medicine, Beni-Suef University, Beni Suef, Egypt

**Keywords:** Melanocytoma, Meningeal, Spinal tumors, Pediatric, Melanoma

## Abstract

**Background:**

Meningeal melanocytoma is considered a rare lesion arising from leptomeningeal melanocytes. Nearly two thirds of meningeal melanocytomas were reported in the intracranial compartment and the remaining one third in the spine. Spinal melanocytomas can be extradural or intradural, with extradural variant being more common, and the majority of cases have been single reports.

**Methods:**

A 5-year-old male presented with a 4-month history of non-radiating low back pain persistent at rest, with otherwise non-remarkable medical history. The patient was neurologically intact with no deficits. Preoperatively, routine laboratory investigations were non-remarkable. MRI imaging was done and showed a lesion at the level of T11 to L4, hyperintense on T1 and hypointense on T2 with homogenous contrast enhancement. Intraoperatively, the lesion was hemorrhagic, brownish, and rubbery in consistency attached to the ventral dura. Microscopic picture revealed dense cytoplasmic brown melanin pigments, with no significant mitoses or nuclear atypia. What is unique about our case is the age of the patient (5 years).

**Results:**

To the best of our knowledge, after reviewing the literature, this is the youngest case to be reported.

**Conclusions:**

SMM is an extremely rare tumor with a benign course. Complete surgical excision should be attempted. Age of presentation may be as young as in our case and the diagnosis of such a tumor should never be excluded in this early age group with persistent low back ache.

## Background

Meningeal melanocytoma is considered a rare lesion arising from leptomeningeal melanocytes. It is the benign pole of the spectrum of neoplastic leptomeningeal melanocyte proliferations that involves primary malignant melanoma at the other pole, from which it differs in behavior and aggressiveness by owing a much better prognosis than its malignant variant (Painter et al. 2000). They may arise anywhere in the cranial and spinal meninges. Two thirds of meningeal melanocytomas were reported in the intracranial compartment and the remaining one third in the spine (Wang et al. 2013). Though rare, local recurrence or leptomeningeal spread secondary to malignant transformation has been reported years after the initial diagnosis (Wang et al. 2007). Spinal melanocytomas can be extradural or intradural, with extradural variant being more common. With around 60 cases reported in the literature, the majority of cases have been single reports (Mangels et al. 2006). We present this case being the youngest patient diagnosed with spinal meningeal melanocytoma. Written informed consent was obtained from the patient for publication of this case report and accompanying images.

## Case description

**Fig. 1:**
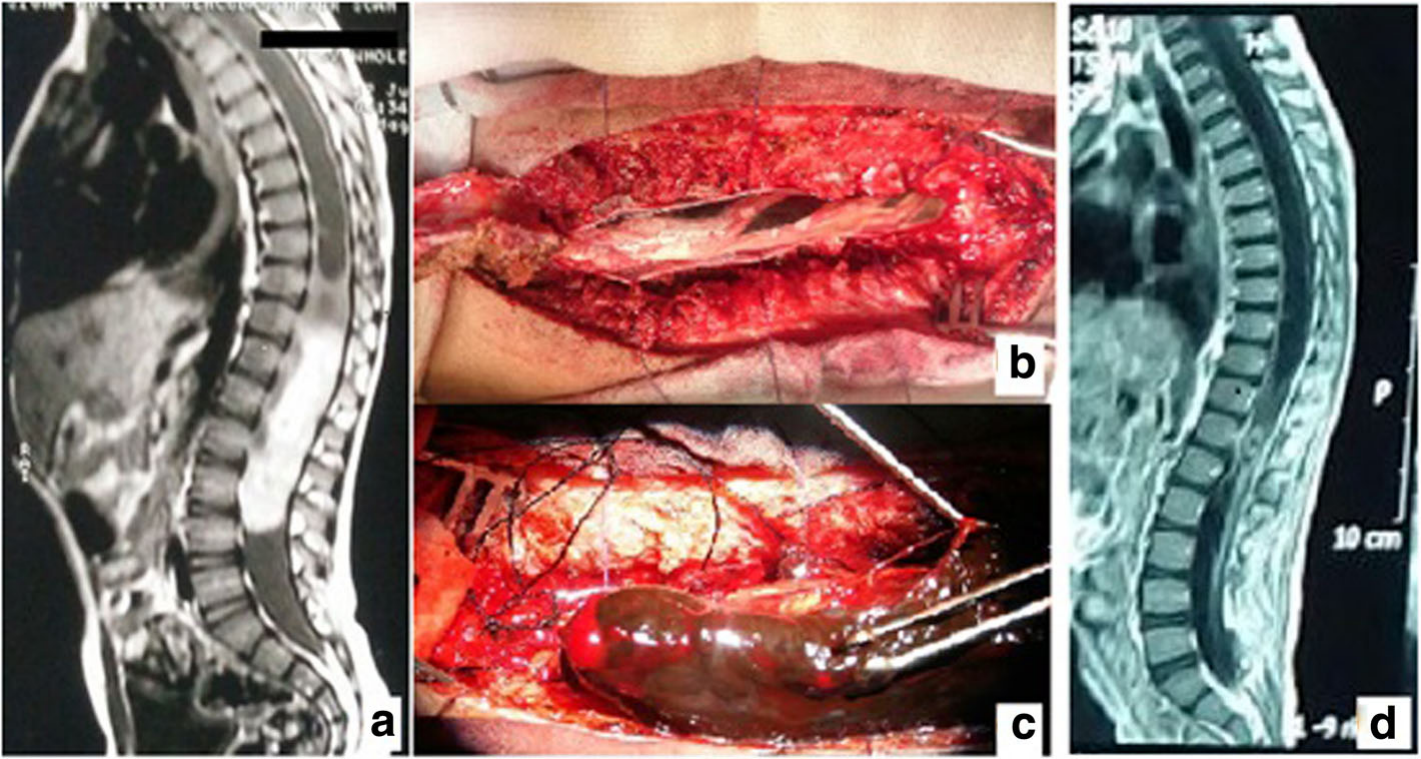
**a** Preoperative MRI T1WI with contrast. **b** Intraoperative view with the lesion under the nerve roots. **c** During tumor removal. **d** Postoperative MRI T1WI with contrast

A 5-year-old male came to our outpatient clinic presenting with a 4-month history of non-radiating low back pain, with otherwise non-remarkable medical history. He was referred to us from a pediatrician after failed medical treatment. By clinical examination, the patient was neurologically intact with no deficits. The persistence of pain during rest and at night raised our suspicion. So, we decided to perform MRI. The preoperative MRI images (using a 1.5 Tesla MRI scanner Signa Hdxt, GE Healthcare, USA) showed a lesion involving the distal dorsal cord as well as the conus medullaris and the cauda equina roots at the level of T11 to L4 averaging 9 cm in long axis. The lesion was hyperintense on T1 with isointense signal at the cephalad end; in T2, it was hypointense and it showed intense homogenous contrast enhancement. There was a small syrinx proximal to the lesion, with no peritumoral edema (Fig. 1a). Preoperatively, routine laboratory investigations were non-remarkable. The patient was operated upon in prone position. A midline low back incision was done followed by sub-periosteal muscle separation and laminoplasty using a bilateral gutter technique (leveling intraoperative was done by fluoroscopy). The dura was then opened in midline and dural tack-up sutures were done. At this point, a brownish black space-occupying lesion was seen among the roots attached to the ventral dura (Fig. 1b). The lesion was adherent but dissectible from cauda equina roots (Fig. 1c). The lesion was totally excised except for a small stump which was adherent to the conus medullaris. Proper hemostasis was done, dura was closed in a water-tight fashion, laminae were replaced using silk sutures, and a subfascial drain was used for 2 days. Postoperatively, the patient was neurologically intact. On the fourth day postoperatively, the patient started to develop suprapubic swelling with decreased frequency of micturition. A Foley catheter was inserted. Postoperative MRI images were done (using a 1.5 Tesla MRI scanner Intera, Philips Medical systems, Best, Netherlands), which showed the small residual left at the conus medullaris (Fig. 1d). Three weeks later, the patient started to feel the desire of micturition with frequent bladder training and the catheter was removed. The patient was discharged with a good general condition.

**Fig. 2:**
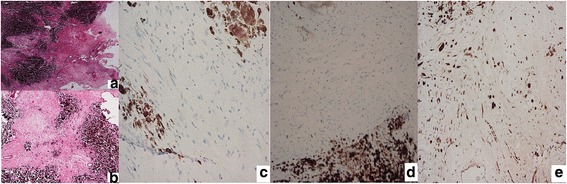
Images **a** (× 40) and **b** (× 100) are hematoxylin and eosin-stained slides showing tumor formed of epithelioid and spindle cells mostly masked by dense brown pigment deposition. Immunohistochemistry: negative for epithelial membrane antigen (EMA) (**c**). Less than 1% of tumor cells are stained for Ki67 (**d**). Tumor cells are focally positive for S100 (**e**)

Grossly, the lesion was irregular, hemorrhagic, brownish black, and rubbery in consistency, measuring 7.5 × 3 × 2 cm. Microscopic picture using hematoxylin and eosin stains revealed hypo- to moderately cellular tumor composed of spindle cells with bland oval nuclei with some macrophages and dense cytoplasmic brown melanin pigments, with no significant mitoses or nuclear atypia (Fig. 2a × 40, Fig. 2b × 100). Immunohistochemistry: negative for epithelial membrane antigen (EMA) (Fig. 2c). Less than 1% of tumor cells are stained for Ki67 (Fig. 2d). Tumor cells are focally positive for S100 (Fig. 2e).

## Discussion and evaluation

Melanocytoma is a pigmented neoplasm of the meninges, which arises from melanocytes derived from the neural crest (Turhan et al. 2004). It is a rare tumor and was first described in 1972 by Limas and Tio (1972). It usually occurs in the spine and posterior fossa (Shanthi et al. 2010). What is unique about our case is the age of the patient (5 years). To the best of our knowledge after reviewing the literature, this is the youngest case to be reported (Clarke et al. 1998). The usual age of presentation is usually around the fifth decade (Czarnecki et al. 1997; Hou et al. 2012), with age ranging from 9 to 79 years in different case reports reviewed (Shanthi et al. 2010; Clarke et al. 1998; Czarnecki et al. 1997; Hou et al. 2012; Alameda et al. 1998). In the study of Yang et al. (2016) (the largest case series in literature) which included 15 patients from the period of 2004–2014, the youngest patient was 14 years. The youngest case reported before our case was 9 years in the study of Alameda F. et al. in 1998 (Alameda et al. 1998).

As for clinical presentation, our case had only a 4-month history of non-radiating low back pain with no radiculopathy or sphincter affection. In literature, the most common feature was radiculopathy, either pain or heaviness followed by back pain and sphincter affection (Czarnecki et al. 1997; Hou et al. 2012; Yang et al. 2016; Dorwal et al. 2014; Sen et al. 2011).

For spinal meningeal melanocytoma (SMM), it is usually intradural extramedullary and to a lesser extent intramedullary (Of all 60 SMM, 40 cases were intradural extramedullary and 19 cases were intramedullary.) and this goes along with our case, with cervical levels being affected more than dorsal levels, and the lumbar levels were the least (Clarke et al. 1998; Hou et al. 2012; Alameda et al. 1998; Eun et al. 2011).

As for the radiological appearance, it correlates well with the available literature (Hou et al. 2012; Alameda et al. 1998). The signal intensity in different sequences depends mainly on the concentration of the melanin pigment in the tumor and so does the contrast enhancement. Lesions with high melanin content may show subtle contrast enhancement on T1WI with contrast as it may be masked by the hyperintensity of melanin (Alameda et al. 1998).

Surgical resection is the main choice for treatment of SMMs. These are benign lesion which can be excised totally whether being intradural extramedullary (possibly en bloc) or intramedullary lesions (Yang et al. 2016; Horn et al. 2008). Our lesion was subtotally resected due to the intermingled part with the conus medullaris. Local control by radiotherapy is advisable in case of incomplete excision, as recurrence may transform into malignant melanoma (Rades et al. 2004). Even in complete resection, some authors recommend radiotherapy, as recurrence was reported in 22% of cases at 5 years (Rades and Schild 2006). However, we decided not to administer radiotherapy and to follow the lesion with serial imaging studies, owing to the benign nature of the lesion and the young age of the patient who could be harmed by the potential adverse effects of radiotherapy on a young age. Stereotactic radiosurgery is another acceptable alternative with a favorable outcome after incomplete resection (Ali et al. 2009).

## Conclusions

SMM is an extremely rare tumor with a benign course. Surgical excision in an en bloc manner should be the model. The outcome is generally good with complete remission and long-term free survival. Age of presentation may be as young as in our case and the diagnosis of such a tumor should never be excluded in this early age group with persistent low back pain.

## Abbreviations

EMA: Epithelial membrane antigen
SMM: Spinal meningeal melanocytoma
